# Determinants of unwanted pregnancies in India using matched case-control designs

**DOI:** 10.1186/1471-2393-12-84

**Published:** 2012-08-11

**Authors:** Priyanka Dixit, Faujdar Ram, Laxmi Kant Dwivedi

**Affiliations:** 1 International Institute for Population Sciences, Mumbai, India; 2 Tata Institute of Social Sciences, Mumbai, India

## Abstract

**Background:**

In India, while the total fertility rate has been declined from 3.39 in 1992–93 to 2.68 in 2005–06, the prevalence of unintended pregnancy is still stagnant over the same period. A review of existing literature shows that within the country, there are variations in fertility preferences between different regions. Also there is a strong argument that the availability of a health facility at the village level plays an important role in reshaping the fertility behavior of women. Keeping in mind the fact that there is no information at the village level (which is the lowest geographical boundary) in the recent round of National Family Health Survey (NFHS-3), the specific objective of this study is to examine the impact of individual and household level variables on unwanted pregnancies without controlling the village level variation. Further, once the village level variation (i.e. unobserved variation) has been controlled, it is necessary to study whether there has been any alteration in the contribution of factors from earlier results of without adjusting the village level variation.

**Methods:**

This paper attempts to examine the associated factors of unwanted pregnancies, without matching the village and after matching the village, by using the matched case–control design. Nationwide data from India’s latest NFHS-3 conducted during 2005–06 was used for the present study. Frequency and pair wise matching has been applied in the present paper and conditional logistic regression analysis was used to work out the models and to find out the factors associated with unwanted pregnancies.

**Results:**

A major finding of this study was that 1:3 case–control study (without matching the village) shows that women belonging to non Hindu/Muslim religion, Scheduled Tribe, women who have experienced child loss and if the previous birth interval is 24 through 36 months were significant predictors of unwanted pregnancy. However, this relationship did not hold significant after village wise matching. Other factors such as Muslim religion, women and their partners with high school education and above, women belonging to the richest wealth index and if the sex of the last child was female, emerge as significant predictors of unwanted pregnancies.

**Conclusions:**

This study clearly underscores the importance of adjusting the village (PSU) level variation in explaining unwanted pregnancies.

## Background

The ability of couples to plan the number, spacing and timing of births is an important fundamental human reproductive right. Although total fertility rates are helpful in estimating the effect of demographic goals and population policies, they do not shed much light on the extent to which individual women exercise their right to decide when they want to get pregnant. In this regard, pregnancy intention seems to be an accurate indicator. Previous literature shows that unwanted pregnancies are not an uncommon phenomenon. In many developing countries, births that women have but do not want constitute a substantial proportion of all births [1-6]. Moreover, in India, while the total fertility rate has declined from 3.39 in the period 1992–93 to 2.68 in 2005–06, the prevalence of unintended pregnancy (both unwanted and mistimed) has been stagnant over the same period. About one-fourth of the women reported that their pregnancy was unintended in all three rounds of National Family Health Surveys [7-9] (IIPS & Macro International, 2007; 2000; 1995). This clearly shows that unwanted pregnancy currently poses one of the greatest challenges associated with Indian women's reproductive health.

Unintended pregnancy is an important public health issue in developing countries like India because of its association with adverse social and health outcomes. Studies conducted in various developed and developing countries revealed that unintended pregnancies can have serious health, social, and economic consequences [10-12]. It can have negative economic, educational and social consequences for both the family and the nation. Unintended pregnancy may be associated with unhealthy behavior before, during and after pregnancy and can adversely affect pregnancy outcomes. Several studies have shown that unwanted fertility has unfavorable effects on antenatal, postnatal preventive and curative care. Women who experience an unwanted pregnancy are less likely to receive care than women who had an intended pregnancy [13,14]. The negative consequences of unwanted pregnancies are increased risk of low birth weight and of being born prematurely; as a result, infants have a high risk of mortality. Many researchers have assessed the effect of unintended pregnancies on place of delivery, child immunization and breastfeeding behavior [15,16].

Reduction in the level of unwanted pregnancy has important social, health and demographic consequences. At the individual level, preventing unwanted birth enhances the well being of women and their children. At the societal level, eliminating unwanted births leads to substantial reduction in fertility and the rate of population growth [1]. Unintended pregnancy poses significant public health risks. In fact, the reduction of unwanted fertility may be a key to reducing maternal and child mortality and to reaching Millennium Goals 4 and 5.

The study of unwanted childbearing sheds considerable light on the reproductive process. However, a complete picture of the factors associated with unwanted pregnancies can be gleaned with the help of important covariates like place of residence, age of women, number of living children, child loss, preceding birth intervals, and measure of economic status [17,18]. A study stated that the prevalence of unwanted births typically increases with age and parity because women who have already achieved their desired family size do not want any more pregnancies [12]. Individual level covariates such as, interval from last live birth to index pregnancy, ever contraceptive use, ever physically mistreated by husband were found to be significant factors associated with unwanted pregnancies [19]. Moreover in low-income countries, less proportion of couples using contraception continued to be the main factor influencing the prevalence of unintended pregnancy [20]. This study maintains that in the Indian situation, socio-cultural and environmental factors play a major role in understanding the factors affecting unwanted pregnancies. However, the variables related to these factors are not available in large scale surveys like NFHS-3, but can be captured with the help of “region (village)” variable.

Several studies suggest that within the country, variation in fertility preferences exist among regions [1,7]. From the sociological point of view, social structure and culture shape individual behavior and provide the circumstances within which individuals live. That is, individuals have the option to exercise his/her choice within the socio-cultural context in which they live. Under the assumption that culture varies across regions, the region in which an individual currently lives may be a significant determinant of a variety of behavior. A state specific analysis in India shows that within country variation in unwanted fertility is much higher than variation in wanted fertility [21]. However, in the Indian situation, the lowest geographical unit is the village which has different socio-cultural and environmental conditions. Against this background, studies underscore the role of the village in explaining various demographic indicators including unwanted births [22,23].

There is a strong argument that availability of health facilities at the village level plays an important role in reshaping the fertility behavior of women [24]. Keeping in mind the fact that there is no information at the village level (which is the lowest geographical boundary) in the recent round of National Family Health Survey (NFHS-3), the specific objective of this study is to examine the impact of individual and household level variables on unwanted pregnancies without controlling the village level variation. Further, once the village level variation (i.e. unobserved variation) has been controlled, it is necessary to study whether there has been any alteration in the contribution of factors from earlier results of without adjusting the village level variation. In this situation, the variable, ‘village’ (also known as primary sampling unit (PSU) in NFHS) is a substitute for many important indicators (observed as well as unobserved) including the level of health care offered in the village, which plays a vital role in explaining the variation in unwanted pregnancy.

## Methods

### Study population

Nationwide data from India’s latest NFHS-3 conducted during 2005–06 was used for the present study. The main objective of the survey is to collect reliable up to date information on maternal, child health and family planning and to provide state level estimates. This survey covered a representative sample of 124385 women in the age group of 15–49 years. The sampling method used under NFHS was multistage systematic random sampling. NFHS-3 collected information of the status of last birth which occurred during the five years preceding the survey [7]. Among 124385 women, only 36832 women experienced at least one birth during the five years preceding the date of survey. The wanted, mistimed and unwanted pregnancies were reported by 78.9 percent, 10.5 percent and 11.4 percent of women, respectively.

### Variables

Pregnancy intention is determined by the mother's response to the question, "Going back to just before you got pregnant, how did you feel about becoming pregnant?" If women reported that she wanted to be pregnant, then she was considered to have had an intended pregnancy. A woman who reported that she wanted to be pregnant later was considered to have had a mistimed pregnancy and a woman who did not want to be pregnant at that point of time or at any time in the future, was considered to have had an unwanted pregnancy. In this study, the dependent variable is the reported unwanted pregnancy. Earlier studies show that mistimed and unwanted pregnancies have different risk factors, and the circumstances in which they occur are also different [25-28]. In this regard 3848 women, who have reported mistimed pregnancy, have been excluded from the analysis.

Although a number of potential explanatory variables were initially considered for the analysis, variables such as working status of women, partner’s occupation and exposure to mass media have been included before finalizing the variables. Only a few meaningful ones were included in the model owing to the lack of association with the outcome variable followed by preliminary analysis. The final list of selected explanatory variables for the present study is:

Community level variables like region (central/north/east/northeast/west/south), place of residence (rural/urban), household level variables such as wealth index (poorest/poorer/middle/richer/richest) which is a proxy for the socioeconomic status of women, religion (Hindu/Muslim/Others) and caste (Scheduled Caste/Scheduled Tribe/Others) have been considered. Individual-level variables included were woman’s education (illiterate/literate but below primary/primary but below middle/middle but below high school/high school and above), partner’s education (illiterate/literate but below primary/primary but below middle/middle but below high school/high school and above), sex of last child (male/female); ever used contraceptive (never used/ used); sex composition of living sons and daughters (number of sons greater than number of daughters/number of sons less than number of daughters/ number of sons equal to number of daughters); whether experienced child loss (none/1+) and previous birth interval (first birth/ less than 24 months /24 to 36 months / more than 36 months).

### Statistical analysis

To examine the factors associated with unwanted pregnancies by using matched case–control design the following steps were performed:

1. Selection of case and control

2. Matching

3. Analysis

#### Selection of case and control

A case–control study can be restricted to any (sub) type of case that may be of interest. A recently matched case control design has been applied in cross sectional data to investigate the relationship between youth assets and sexual intercourse among 13–14 year olds [29]. In a matched case–control study, the cases emerge from a well-defined population and the controls are also sampled from the same population. In this analysis, a case is defined as any woman who experienced unwanted pregnancies during the five years preceding the date of survey and their counterparts were selected as controls. The choice of a control group was entirely determined by the definition and selection of the case group. A maximum number of three controls were available for this study.

#### Matching

Matching refers to the selection of controls that are similar to cases with respect to the distribution of one or more potential confounding factors. Potential confounding variables for a case–control study should be selected a priori using literature relevant to the target population, rather than by statistical criteria among participating cases and controls [30]. The advantage of matched case control design over unmatched design is that if the distributions of a confounder are substantially different in cases and controls, matched case–control studies control for confounding by introducing stratification in the study design [31,32]. Matched study can increase the efficiency of the study by balancing strata which overcomes sparse-data problem and it gives the maximum information even sample size is small.

Two types of matching schemes are commonly used, frequency (category/group) matching and pair matching. Both types of matching have been performed in the present study. To analyze the determinants of unwanted pregnancies, first we have performed the frequency matching taking women’s age as a cofounding variable. Studies in several developing countries illustrate that unwanted pregnancies are strongly associated with maternal age [33-36]. With the help of frequency matching, we are in a position to find out the factors affecting unwanted pregnancy without matching by village.

In the second step to deal with the complex hierarchical inter-relationship between the variables, we have performed paired matching using women’s age and village (which is the lowest geographical boundary) as matching covariates. Finally, pair-wise matched method is useful in explaining the factors affecting unwanted pregnancy after controlling the unobserved heterogeneity at village level. Moreover in the absence of village level information in the recent round of NFHS-3 data, the matching at village level helps in controlling the unobserved variation. It could be observed that important village-level variables like availability of health facilities/all-weather road, and distance to the nearest transport facility differ from one village to another. Moreover, there are certain unobserved factors which surveys do not capture such as motivation and perception of the women towards utilization of health facilities is generally determined by village specific socio-cultural and environmental factors. At the last the aim is also to compare the obtained results with the help of both matched methods.

In frequency matching, age of the women and in pair-wise matching in addition to woman’s age, region of residence (village) were used to match the cases and controls and therefore, matching factors can no longer be investigated in the analysis, as these have been used as stratification variables [31,32,37].

## Analysis

### Frequency matching

Women who have given at least one birth during five years preceding the survey (36,832 women) have been selected for the analysis. In the selected samples, unwanted (4187) births are considered cases. Controls were selected randomly from 28,797 women, who had reported wanted pregnancy. Mistimed births (3,848) were not included in the analysis. Cases and controls were matched for age (five years age group) of women. Further, we have applied 1:3 case–control ratios that is, three controls for each case because of the maximum number of availability of controls for each case [38].

After selection of cases and controls in frequency matched case control design, the two groups of the data are still different except for the matching variable, which satisfy the conditions of unconditional logistic regression model [39]. Previously many researchers have also applied unconditional logistic regression in frequency matched case–control design [33,40]. It is used to identify the influencing factors thought to be the determinant of unwanted pregnancies. The advantage of the unconditional logistic regression procedure is that it models the log of the odds of an outcome occurring in terms of a vector of independent variables. It is defined as

(1) $$\text{log}\;\left(\text{Y}\right)=\text{a}+{\text{b}}_{1}{\text{X}}_{1}+{\text{b}}_{2}{\text{X}}_{2}+{\text{b}}_{3}{\text{X}}_{3}+\ldots \ldots \ldots \ldots .+{\text{b}}_{\text{n}}{\text{X}}_{\text{n}}$$

Where log (Y) is the natural logarithm of the odds of the outcome, ‘a’ is the intercept and b_1_; b_2_; etc. are the coefficients associated with each predictive variable.

### Pair matching

Cases and controls were matched for region of residence (village-primary sampling unit (PSU), which is the lowest geographical boundary) and age of the woman in a five year interval. To make each control similar as case, within a PSU an age wise matching (five year age group) was performed. With the help of PSU matching, we were able to control many socio-cultural, environmental and programme level factors [41]. Around 476 cases were dropped from the analysis because controls similar (in terms of PSU and age) to cases were not available. After matching, total controls were 3711 women, who reported that their last birth was wanted. Following this, 1:1 pair matching was performed. Conditional logistic regression analysis was used to work out the models and to find out the factors associated with unwanted pregnancies. Conditional logistic regression and matched study design can yield increases in efficiency compared to an unmatched design with an unconditional logistic regression approach [31]. In a pair wise matching, region, place of residence and age variables were excluded from the analysis and all other variables remain the same.

After analysing the data, derived coefficients have been interpreted for their significance and transformed through exponentiation to yield odds ratios that indicate the magnitude of the variables impact on the probability of the occurring outcome. The results in this study are interpreted in terms of odds ratios. The statistical analyses were made using a computer programme Stata (version 8.0, Stata Corporation, College Station,) and a p-value less than 0.05 was considered statistically significant.

### Ethical review

This study is based on the National Family Health Survey data. The National Family Health Survey was conducted under the scientific and administrative supervision of the International Institute for Population Sciences, (IIPS) Mumbai, India. The IIPS is a regional center for teaching, training and research in population studies, and is associated with the Ministry of Health and Family Welfare, Government of India. The Institute conducted an independent ethics review of NFHS protocol. Data collection procedures were also approved by the ORC Macro institutional review board. The present study is based on NFHS data which is the public use data set with no identifiable information on the survey participants.

## Results

Table 1 presents the percentage distribution of total unwanted (n = 4187) and wanted births (n = 28797) in relation to selected background characteristics of Indian women in the year 2005–06. The last column of the Table 1 shows the percentage distribution of wanted birth under 1:3 case control approach. In 1:3 case controls, total sample size for controls was 12561 women.

**Table 1:** Percent Distribution of Unwanted and Wanted Births by Selected Background Characteristics under Full Sample Model and 1:3 Case Control Approach

**Explanatory Variables**	**Unwanted (Full Sample)**		**Wanted (Full Sample)**		**Wanted (Control in Ratio 1:3)**	
	**Percentage**	**Sample size**	**Percentage**	**Sample size**	**Percentage**	**Sample size**
**Region**						
Central	42.95	1434	26.14	6429	26.44	2682
North	8.56	491	12.91	4734	13.76	2089
East	27.55	746	24.77	4466	23.82	1787
Northeast	3.12	813	4.17	5154	4.72	2607
West	6.73	233	14.16	3604	13.73	1611
South	11.10	470	17.85	4410	17.53	1785
**Place of Residence**						
Rural	77.78	2721	72.74	17285	71.24	7339
Urban	22.22	1466	27.26	11512	28.76	5222
**Religion**						
Hindu	74.32	2681	79.76	20607	78.34	8703
Muslim	22.10	918	15.26	4333	16.12	1869
Others	3.58	588	4.98	3857	5.53	1989
**Caste**						
Others	70.43	2728	70.4	19604	70.38	8493
Scheduled Caste	21.65	791	19.73	4909	19.32	2028
Scheduled Tribe	7.92	668	9.87	4284	10.3	2040
**Women's Education**						
Illiterate	63.73	2263	46.56	10843	50.28	4913
Literate but below Primary	7.34	378	7.04	2062	6.53	849
Primary but below Middle	11.87	597	14.9	4285	12.52	1603
Middle but below High School	8.51	434	12.21	4169	9.69	1555
High School and above	8.55	515	19.3	7438	20.98	3641
**Partner’s education**						
Illiterate	38.65	1417	27.94	6602	30.73	3017
Literate but below Primary	8.38	387	7.38	2047	7.42	907
Primary but below Middle	15.62	696	15.26	4237	14.37	1712
Middle but below High School	14.81	667	16.87	5142	15.04	2027
High School and above	22.55	1020	32.55	10769	32.46	4898
**Wealth index**						
Poorest	31.19	977	23.72	4705	25.01	2079
Poorer	25.59	903	21.10	4903	20.68	2065
Middle	20.41	931	19.20	5628	17.36	2241
Richer	14.64	811	18.58	6356	17.48	2640
Richest	8.17	565	17.41	7205	19.47	3536
**Sex of last child**						
Male	50.94	2122	54.71	15661	54.86	6862
Female	49.06	2065	45.29	13136	45.14	5699
**Age of the respondent**						
Less than 25 years	15.35	608	43.70	11023	17.74	1824
25-29 years	30.91	1281	32.81	9791	32.04	3843
30+ years	53.73	2298	23.49	7983	50.22	6894
**Ever Used Contraceptive**						
Never Used	31.07	1265	44.66	11930	40.13	4723
Used	68.93	2922	55.34	16867	59.87	7838
**Sex Composition of Living Children**						
No. of Sons Greater than Daughters	42.48	1725	38.77	11096	37.53	4670
No. of Sons Less than Daughter	38.47	1650	38.67	11256	39.53	4984
No. of Sons Equal to Daughters	19.05	812	22.56	6445	22.94	2907
**Experience of Child Loss**						
None	63.94	2874	80.13	23892	74.49	9951
At least one	36.06	1313	19.87	4905	25.51	2610
**Previous Birth Interval**						
First Birth	2.93	183	30.93	9346	19.43	2783
Less than 24 months	23.06	944	15.36	4322	14.85	1779
24 to 36 months	34.29	1361	24.35	6551	25.09	2921
More than 36 months	39.72	1699	29.36	8578	40.64	5078
**Total**	**100**	**4187**	**100**	**28797**	**100**	**12561**

The table reflects that demographic and socioeconomic characteristics such as region, place of residence, religion, caste, women and partner’s education, wealth index, sex of child, sex composition of living children and previous birth interval were associated with unwanted pregnancies. A high proportion of women living in the central region and in rural areas, who were Hindus and belonged to non Scheduled Caste/Tribe, reported more unwanted births. Unwanted births were higher than those in the other categories if either the woman or her partner was illiterate. The analysis shows that as the wealth index increases, the percentage of unwanted pregnancies decreases. The proportion of unwanted pregnancies has a positive association with the age of women. Regarding reproductive characteristics of women, results indicate that a slightly higher proportion of women had more number of sons than daughters and that most of the women reported that their last child was male. Of total 4187 women, most respondents reported that they had not experienced child loss.

Adjusted odds ratio of unwanted birth using multiple unconditional logistic regression analysis under 1:3 case control approach is given in Table 2. Last panel of Table 2 also showed the adjusted odds ratio of unwanted births by conditional logistic regression under case control approach and its 95 percent confidence interval (CI). This table displays the adjusted odds ratio and its 95 percent CI estimate with respective unwanted pregnancy under 1:3 case control and conditional logistic approach in various categories of a variable in comparison to the reference category which was adjusted for region, place of residence, religion, caste and also for the remaining considered variables.

**Table 2:** Adjusted Odds Ratio (with 95% Confidence Interval) of Unwanted Pregnancy Using Multiple Unconditional Logistic Regression and Conditional Logistic Regression Analysis under Case Control Approach

**Explanatory Variables**	**Case Control Approach in the Ratio of 1:3**			**Pair Wise Matching Case Control Approach**		
	**EXP (B)**	**95% C.I. EXP (B)**		**EXP (B)**	**95% C.I. EXP (B)**	
		**Lower**	**Upper**		**Lower**	**Upper**
**Region**						
Central®						
North	0.50	0.44	0.56			
East	0.78	0.70	0.88			
Northeast	0.62	0.55	0.71			
West	0.35	0.30	0.41			
South	0.62	0.54	0.71			
**Place of residence**						
Rural®						
Urban	1.12	1.02	1.24			
**Religion**						
Hindu®						
Muslim	1.37	1.24	1.52	1.27	1.02	1.60
Others	1.27	1.10	1.46	1.11	0.83	1.50
**Caste**						
Others®						
Scheduled Caste	1.06	0.95	1.17	0.96	0.82	1.13
Scheduled Tribe	0.87	0.76	0.98	1.02	0.78	1.34
**Women's Education**						
Illiterate®						
Literate but below Primary	1.16	1.00	1.33	1.10	0.90	1.36
Primary but below Middle	1.05	0.93	1.19	1.01	0.85	1.20
Middle but below High School	0.92	0.80	1.06	0.91	0.74	1.12
High School and above	0.62	0.53	0.72	0.80	0.64	1.00
**Partner’s education**						
Illiterate®						
Literate but below Primary	0.98	0.85	1.13	1.14	0.93	1.41
Primary but below Middle	0.95	0.85	1.07	0.98	0.82	1.16
Middle but below High School	0.86	0.76	0.97	0.76	0.64	0.91
High School and above	0.85	0.75	0.97	0.81	0.68	0.97
**Wealth index**						
Poorest®						
Poorer	1.01	0.89	1.13	0.85	0.72	1.01
Middle	1.07	0.95	1.22	0.83	0.68	1.01
Richer	0.91	0.79	1.05	0.69	0.55	0.87
Richest	0.71	0.59	0.85	0.56	0.42	0.74
**Sex of last children**						
Male®						
Female	1.33	1.22	1.45	1.46	1.29	1.64
**Ever Used Contraceptive**						
Never Used®						
Used	1.55	1.43	1.69	1.85	1.64	2.09
**Sex Composition of Living Children**						
No. of Sons Equal to Daughters®						
No. of Sons Greater than Daughters	0.77	0.70	0.85	1.70	1.47	1.96
No. of Sons Less than Daughters	0.61	0.55	0.68	1.21	1.05	1.40
**Experience of Child Loss**						
None®						
At least One	1.10	1.01	1.20	1.06	0.94	1.20
**Previous Birth Interval**						
First Birth®						
Less than 24 months	5.92	4.96	7.07	0.14	0.11	0.17
24 to 36 months	5.05	4.26	6.00	0.96	0.83	1.11
More than 36 months	3.91	3.31	4.62	0.82	0.71	0.94

**Note**: ® shows reference category.

A closer look reveals that region and place of residence were found to contribute significantly towards unwanted births. As compared to the central region, unwanted births were less likely in all other region, also those women who were living in urban areas reported more unwanted births. The adjustment demonstrated an insignificant association of intention to have the last child in relation to Scheduled Caste (odds ratio = 1.06, 95% CI 0.97-1.17), education (women literate but below primary, odds ratio = 1.16, 95% CI = 1.00-1.33) (women primary but below middle, odds ratio = 1.05, 95% CI = 0.93-1.19) (middle but below high school, odds ratio = 0.92, 95% CI = 0.80-1.06), (partner literate but below primary, odds ratio = 0.98, 95% CI = 0.85-1.13) (partner primary but below middle, odds ratio = 0.95, 95% CI = 0.85-1.07) and wealth index (poorer, odds ratio = 1.01, 95% CI = 0.89-1.13) (middle, odds ratio = 1.07, 95% CI = 0.95-1.22), (richer, odds ratio = 0.91, 95% CI = 0.79-1.05). If a woman and her partner were educated up to the high school level and above, they were less likely to experience unwanted births. After adjustment, this table clearly indicates that women whose last child was female and who suffered from at least one child loss and used contraceptives ever, were more likely to report that their last birth was unwanted.

After controlling other factors in the logistic regression model result shows that if the child was female (odds ratio: 1.33) and if women ever used contraceptive (odds ratio: 1.55), then they were more likely to report unwanted birth. The odds of having unwanted births for those parents who had lost one child was also higher than those who did not experience any child loss.

The use of region of residence as a control variable is pervasive in demographic research particularly on fertility analysis [42,43]. Previous analysis shows that the community (region and place of residence) where the respondent lives plays a significant role; therefore, to disentangle the relative importance of clustering at different levels and to assess the respective role of individual and socio-demographic factors on unwanted pregnancy among ever married women, pair wise matching has been applied. After that, conditional logistic regression under case control approach has been performed. In this model, region, place of residence and age variables were excluded and all the other covariates retained in the analysis. Each case was matched with control in relation to the age of women (five years age-group) and village where the women were living.

The result based on unadjusted odds ratio of conditional logistic regression along with 95% confidence interval (CI) demonstrates that (table not shown), wealth index, sex of child, experience of child loss, ever used contraceptives were the most important predictors for unwanted birth. However, other variables like, other than Muslim and Hindu religion, caste, women and her partner’s education (literate but below primary, primary but below middle), number of sons less than daughters and if previous birth interval was 24–36 months were not found to be statistically significant.

The results of conditional logistic regression displayed in the last panel of Table 2 showed the adjusted odds ratio of unwanted births and its 95 percent confidence interval in relation to selected covariates. Analysis clearly reveals that Muslim women had 27 percent higher chance of having unwanted pregnancy. Women with high school education and above and whose partners had a similar level of education had about 20 percent less chance of having unwanted pregnancy. If the partner was educated up to middle school but below high school (odds ratio = 0.76, 95% CI 0.64 – 0.90), it still had a significant negative impact on unwanted pregnancy as compared to illiterate partners. Significant wealth index differentials in unwanted pregnancy were evident among richer women (odds ratio: 0.69), but were more glaring among richest women (odds ratio: 0.56). The matching variable village has been used to control the unobserved heterogeneity at the regional level that might be correlated with unwanted pregnancy. After controlling village level and age wise variation, variables such as women belonging to non Hindu/Muslim religion, Scheduled Tribe, women who experienced child loss and if the previous birth interval was 24 to 36 months, have become insignificant. The adjustment again demonstrates the significant association of intention to have the last child along with sex of last child, sex composition of living children and previous birth interval (except previous birth interval was 24 to 36 months). After controlling others variables, Table 2 clearly indicates that those women who were ever users of contraceptives were more likely to report unwanted birth. Women, who have more number of sons than daughters and fewer numbers of sons than daughters, reported 70 percent and 21 percent more likely unwanted birth respectively, as compared to women having equal number of sons and daughters. Women whose previous birth interval was less than 24 and more than 36 months reported less unwanted births compared to the reference category (first birth order).

## Conclusions and discussions

In recent years, unwanted pregnancy emerge as an important public health concern in both developed as well as developing countries because it is not only distressing for the affected women and children, but can also have far-reaching health, social and economic consequences. It is crucial to identify the determinants of unwanted pregnancy to facilitate policy makers and programme managers to design programmes and services particularly for those women who have the highest likelihood of having unwanted pregnancy. In this paper, we applied case control design to identify the various factors that influence the intention to have or not to have the last child of ever married women in the age group, 15–49 years, which acts as an important component of population change. We examine the associated factors of unwanted pregnancies by using matched case–control design. In the data set, distribution of confounding variables is substantially different in case and control groups, so matching is an option that can improve efficiency in the estimation of the effect of exposure. Also, matching with community of residence (village) covers measured and unmeasured confounder variables, may serve as proxies for environmental and socio-economic condition of the village.

In order to highlight the importance of independent factors that influence the issue of unwanted pregnancies, both unconditional and conditional logistic regression analysis in the form of case control design has been performed. From the foregoing discussion of 1:3 frequency case–control design it may be concluded that, a high incidence of unwanted pregnancy was found among central living, urban women, Muslims and non Hindu/Muslim religion, ever users of contraceptives, with female child and those women who had experienced child loss.

The significant differences in intendedness that appeared by region of residence suggest that family planning services need to be expanded or improved in the central region. Such regional disparities may be due to cultural factors as well as to differences in the availability and quality of family services.

Analysis found that residents of rural areas independently lowered the likelihood of unwanted pregnancy relative to urban residence. Contradictory to this, analysis based on Demographic Health Surveys conducted in South American countries suggest that rural women are more likely than urban women to experience unintended pregnancies [44]. This perhaps is because rural women's ideal family sizes are usually higher compared to those of urban living women. In addition to this, in urban areas living space is limited, the cost of living is higher, and these are some possible reasons why urban women were more likely to report the last birth as an unwanted.

The positive association noted in this study between Muslim religion and unwanted pregnancy has also been demonstrated in other studies including those from India, Nepal and Sri Lanka [45-48]. All over the world and across most of the settings, Muslims have more children, are more likely to want another child, and, if they want no more children, are consistently less likely to be using contraception. Moreover Muslim communities are characterized by less autonomy for women and greater pronatalism. According to an Indian national survey, family planning adoption rate was significantly low among Muslim as compared to Hindu women [49]. This suggests that Muslim women need special attention because they are exposed to a higher risk of unwanted pregnancy than Hindu women.

Education decreases the odds that a pregnancy is unwanted rather than wanted [50]. Women who had middle but below high school education, were 13 percent and with high school and above educated were 36 percent less likely than those who had no education to have experienced an unwanted pregnancy. A multinational study revealed that an unwanted pregnancy reduced with the length of education [36]. Literate women were more aware of the value of a small family and of contraceptive methods than illiterate women. Owing to the lack of awareness and knowledge about contraceptive methods, illiterate women were more likely to experience unwanted pregnancy. Therefore, illiterate women had the risk of not using any family planning methods that was many times higher than that by literate women [51]. Current research reveals that unwanted fertility was not only related to the socio-demographic characteristics of women but also to their husband’s level of education.

Women who belonged to the richer and richest wealth quintiles were less likely to report unwanted pregnancies than women from poorer households. Studies conducted in other countries have demonstrated that poverty is statistically related with the rates of unwanted births [52,53]. The poorest women are least likely to be able to pay for family planning services. However, wealthier women typically want smaller families and seek out and use available services. A study based on 10 DHS countries, shows that most of the free or subsidized modern contraceptives were distributed among those people who lived above the poverty line [54].

An analysis of 1:3 case controls study shows a significant positive relationship between experiences of previous child loss and unwanted pregnancy. The findings clearly imply that women were scared of child death and opted for a higher number of children against their desired number and thereby had more unwanted births. The findings reveal that ever users of contraceptive method were more likely than never users to say that the pregnancy had been unwanted than to report that it was wanted. This positive association has also been found in a recent study from India which shows that women who used contraceptives were 36 percent more likely to experience an unwanted pregnancy [19,35]. This could be because of improper use of temporary methods (traditional or modern spacing methods). Users of a method might have higher expectations about limiting or spacing their pregnancies, and thus be more likely to view a pregnancy as unplanned.

One interpretation for this incidence could be that, those women who reported ever use of contraceptives were in an advantageous position in terms of achievement of education, socio-economic position and residents of community with good health facilities. These women become more aware of the desirability of family planning and may come to expect every birth to be an outcome of careful planning. Therefore, there is a higher probability that these women are more conscious about the number of children they would like to have compared to their less educated and less fortunate counterparts. Unfortunately, when these women were not able to use contraceptive methods consistently or discontinued the use of contraceptives and the expectation was not met, the informed women classified pregnancies as unwanted than women who did not consider that childbearing can be under their control.

Numerous studies in India have identified the risk factors of unwanted birth at the national and state levels [19,33,55,56]. However, no studies have yet focused on the risk factors of unwanted pregnancy after controlling village level variation under case–control design. The availability of a health facility at the village level is considered a key issue in women's reproductive rights in India, based on this we carried out conditional logistic regression analysis to identify the factors associated with unwanted births after conducting the matched case–control design. The favourable factors that predicted unwanted pregnancy in the model were Muslim religion, female child, ever used contraceptives, and more number of sons than daughters or more number of daughters than sons, while factors such as richer and richest wealth quintiles, women and partners with high school education and if previous birth interval was less than 24 and more than 36 months, were negatively associated with unwanted pregnancy. With and without controlling unobserved heterogeneity at the PSU level (variation of socio-cultural and environmental factors and also programme variables) a clear difference in the factors associated with unwanted births has been observed in the present study. Matching of village accounts for the differences in the variables, which could not be considered in the data collection. On account of these facts, this procedure has been used to analyze the factors associated with unwanted births. The 1:3 case–control study (without matching the village) revealed that women from non Hindu/Muslim religion, Scheduled Tribes, women who had experienced child loss and if the previous birth interval was 24 to 36 months were significant predictors of unwanted pregnancy. However, the analysis clearly brings out the fact that this relationship was not significant after PSU wise matching (matching with village).

Investigation based on pair wise matching illustrates that women who lived in the same village and belongs to the same age-group, factors such as non Hindu/Muslim religion, Scheduled Tribes and demographic factors such as experience of child loss and where the previous birth interval was 24 to 36 months had become insignificant, though others factors such as Muslim religion, women and their partners with high school education and above, as well as if the sex of the last child was female, again emerged as significant predictors of unwanted birth. One of the probable explanations might be that adequate/inadequate PSU level facility, such as road and health facility within or outside the village/town are important factors for preventing unintended pregnancies rather than some cultural and demographic factors. The significant relationship between sex composition of living children and unwanted pregnancy indicate that, there is a possibility that ideal family size is less compared to actual family size of those women who had more number of sons than daughters, or more number of daughters than sons, but in order to attain their desired sex composition of living children they exceeded their actual family size and reported more unwanted births compared to women who have an equal number of sons and daughters.

Another key finding of pair wise matching is that, after matching village, women belonging to the richer wealth quintile are significantly less likely to have an unwanted pregnancy compared to the poorest women. It may give the idea that women belonging to the richer wealth strata are able to afford the costs of family planning methods compared to the poorest women, therefore they face fewer unwanted pregnancies. This study clearly underscores the importance of adjusting the village (PSU) level variation in explaining unwanted pregnancies.

### Limitations of the study

Some of the limitations of the study should be noted.

The NFHS is a cross-sectional survey that looked retrospectively at woman’s pregnancy intention and its related factors. Our study results should be cautiously interpreted in view of the limitations of the cross-sectional design. Another major limitation of our study is that information was not collected on several factors, such as accessibility and availability of health facility at the village level that might influence the relationship between pregnancy intention status and predictor variables. Although, an attempt has been made to control this information through paired matching design, we are not able to find out the association of these variables with unwanted births. Lastly, this study is based only on unwanted births, but a study of factors affecting mistimed birth is also important for healthy society. Owing to the small size of the sample, we are not able to analyse this component separately.

## Competing interests

The authors declare that they have no competing interests.

## Authors’ contributions

PD, FR and LKD conceptualized the paper and finalized the analysis plan; PD conducted all data analyses and generated the tables; PD prepared the first draft of the paper. FR and LKD have helped in revising the manuscript for intellectual content. All authors read and approved the final manuscript.

## Pre-publication history

The pre-publication history for this paper can be accessed here:

http://www.biomedcentral.com/1471-2393/12/84/prepub
